# The genome sequence of a hydroid, *Candelabrum cocksii *(Cocks, 1854)

**DOI:** 10.12688/wellcomeopenres.22705.1

**Published:** 2024-07-22

**Authors:** Patrick Adkins, Rob Mrowicki, Vengamanaidu Modepalli, Marine Biological Association Genome Acquisition Lab, Darwin Tree of Life Barcoding collective, Wellcome Sanger Institute Tree of Life Management, Samples and Laboratory team, Wellcome Sanger Institute Scientific Operations: Sequencing Operations, Wellcome Sanger Institute Tree of Life Core Informatics team, Tree of Life Core Informatics collective, Darwin Tree of Life Consortium

**Affiliations:** 1 The Marine Biological Association, Plymouth, England, UK

**Keywords:** Candelabrum cocksii, a hydroid, genome sequence, chromosomal, Anthoathecata

## Abstract

We present a genome assembly from an individual *Candelabrum cocksii* (hydroid; Cnidaria; Hydrozoa; Anthoathecata; Candelabridae). The genome sequence is 232.9 megabases in span. Most of the assembly is scaffolded into 15 chromosomal pseudomolecules. The mitochondrial genome has also been assembled and is 14.55 kilobases in length.

## Species taxonomy

Eukaryota; Opisthokonta; Metazoa; Eumetazoa; Cnidaria; Hydrozoa; Hydroidolina; Anthoathecata; Aplanulata; Candelabridae; *Candelabrum*; *Candelabrum cocksii* ( Cocks, 1854) (NCBI:txid264050).

## Background

*Candelabrum cocksii* (Anthomedusae, *Candelabriidae*) is a cnidarian species in the Hydrozoa class ( Cocks, 1854). *Candelabrum* are morphologically unusual hydroids in that they have a vermiform bodyplan. They are typically solitary but can form small pseudo colonies through connected aggregates. They attach to the substrate using simple or modified anchoring filaments with or without perisarc sheath, but without stolons ( de Blainville, 1830). The body of a polyp can be divided into three distinct regions: the foot, the blastostyle region in the middle, and the trunk. The foot, also referred to as the hydrorhiza region, serves the purpose of helping the polyp attach to the substrate. The blastostyle-bearing region is located in the middle of the polyp's body, while the trunk is situated at the distal end. The trunk typically narrows down and features a small circular mouth at the extreme end ( Cocks, 1854). The tentacles of the trunk are usually short and only indistinctly capitate. The tentacle pedicels are highly contractile, and undisturbed animals have long tentacles ( Allman, 1876; Benoît, 1923; Billard, 1921). The genus *Candelabrum* has been found in all of the world's oceans, including at high latitudes in both the northern and southern hemispheres ( Hewitt & Goddard, 2001).

*Candelabrum cocksii* is distributed in the North-Eastern Atlantic region, with its northernmost record being in Norway. It is commonly found on the western coast of Great Britain and France, along the English Channel. The *Candelabrum cocksii* occur at the spring tide low water mark and attached to the underside of large boulders ( Cocks, 1854). *Candelabrum cocksii* is often confused with *Candelabrum phrygium*, however, both species are clearly separable, and they differ in the foot morphology and clasper tentacles are only present in *Candelabrum cocksii* ( Schuchert, 2009). The embryonic development occurs within the embryonic envelope held by the clasper tentacles, which only attach to fertilized eggs capable of forming the necessary envelope ( Beigel-Heuwinkel, 1988). The development results in a young polyp with multiple tentacles, the young polyp has temporary primary tentacles that will be replaced by permanent ones ( Billard, 1921). The genome of *Candelabrum cocksii* represents the first genome of the Candelabridae family. Hydrozoans are a diverse group of cnidarians with complex life cycles. The genome of *Candelabrum cocksii* is expected to shed light on the evolutionary history of hydrozoan characters, including the different stages of the life cycle. This information will be useful for classification purposes, as well as enhancing our understanding of homology, evolutionary loss, and convergence of these characters.

## Genome sequence report

**Figure 1.**
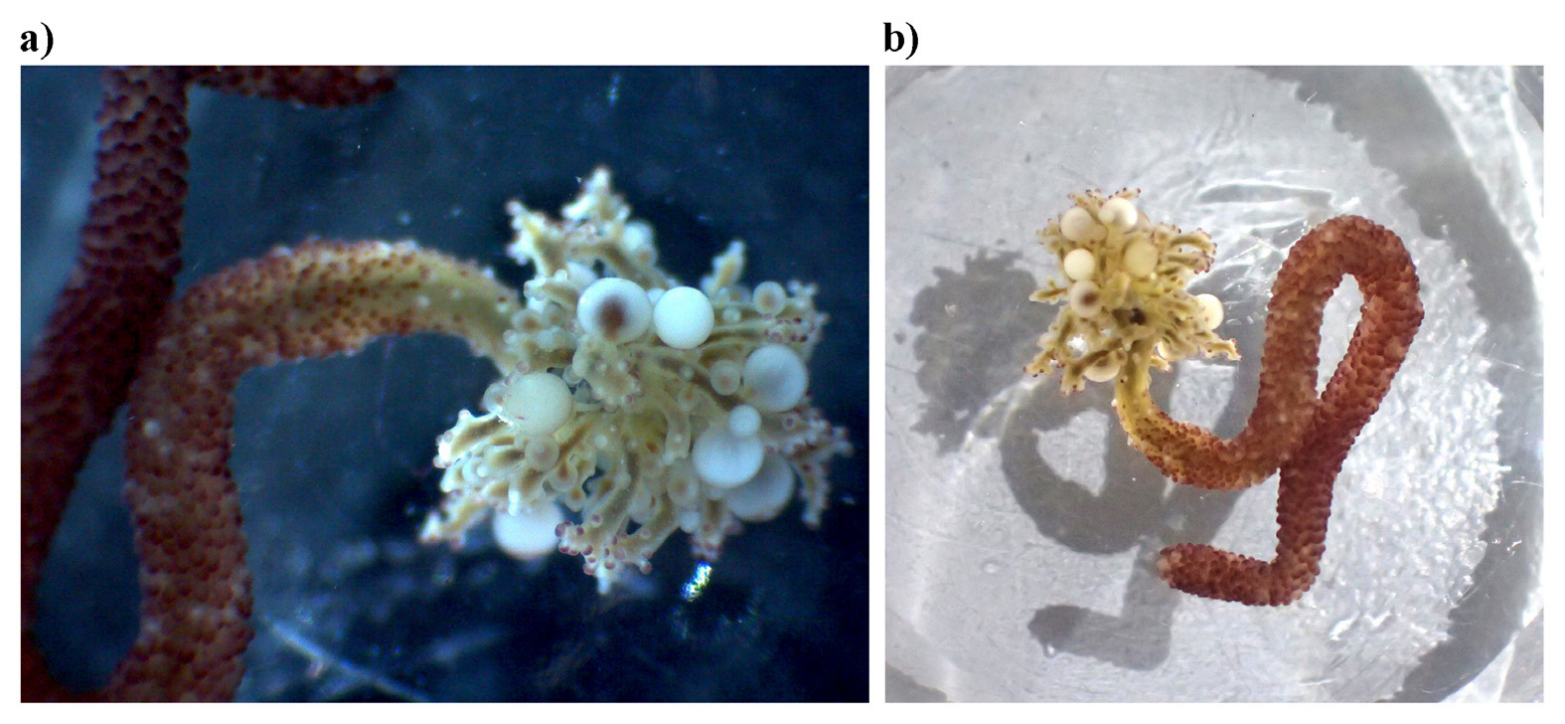
Photographs of the *Candelabrum cocksii* (jhCanCock6) specimen used for genome sequencing.

The genome was sequenced from adult *Candelabrum cocksii* ( Figure 1) collected from Hannafore Point, Looe, Cornwall, UK (50.34, –4.45). A total of 180-fold coverage in Pacific Biosciences single-molecule HiFi long reads was generated. Primary assembly contigs were scaffolded with chromosome conformation Hi-C data. Manual assembly curation corrected 79 missing joins or mis-joins and removed 10 haplotypic duplications, reducing the scaffold number by 50.00%, and increasing the scaffold N50 by 87.35%.

**Figure 2.**
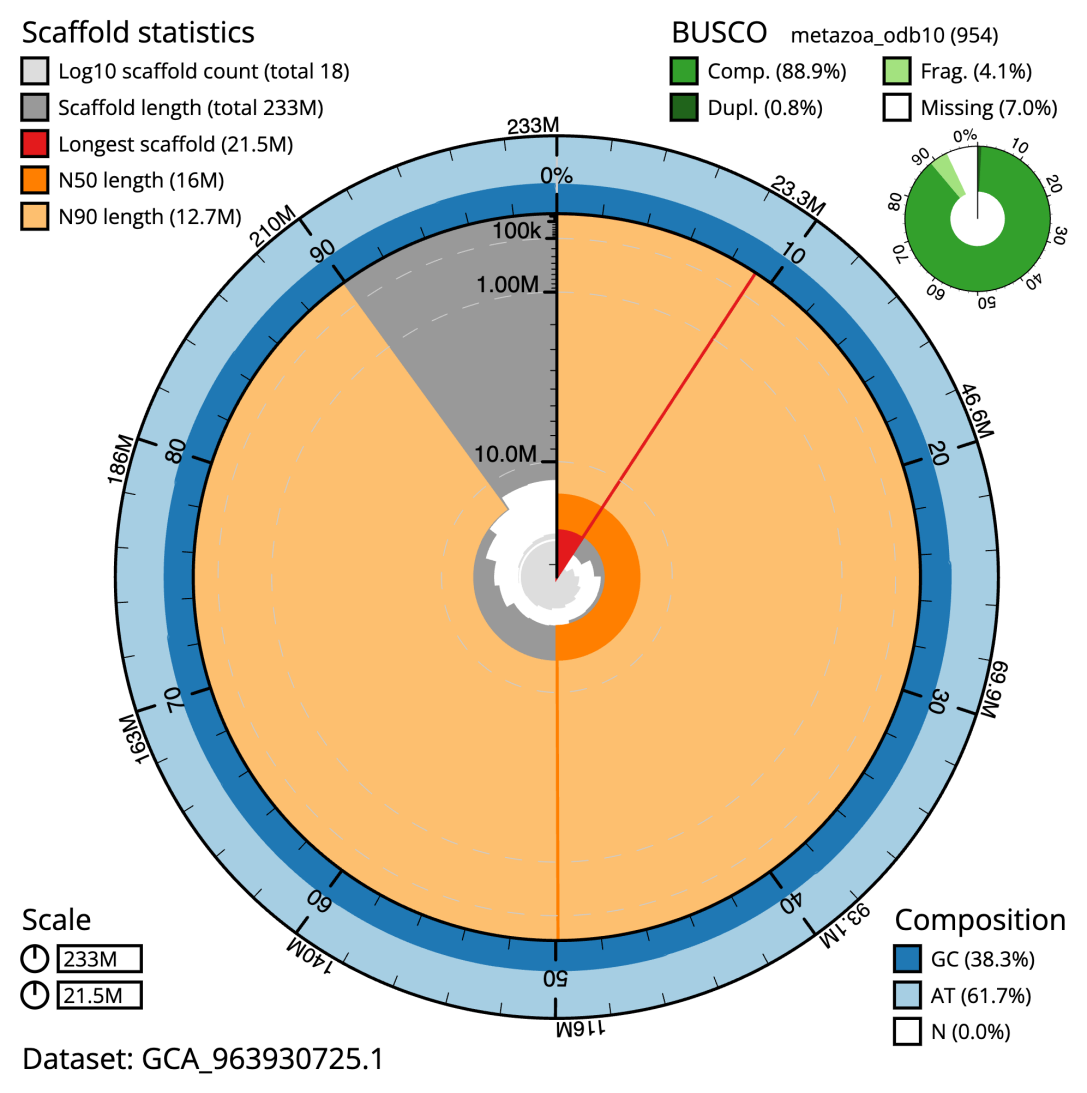
Genome assembly of *Candelabrum cocksii*, jhCanCock6.1: metrics. The BlobToolKit snail plot shows N50 metrics and BUSCO gene completeness. The main plot is divided into 1,000 size-ordered bins around the circumference with each bin representing 0.1% of the 232,868,000 bp assembly. The distribution of scaffold lengths is shown in dark grey with the plot radius scaled to the longest scaffold present in the assembly (21,456,843 bp, shown in red). Orange and pale-orange arcs show the N50 and N90 scaffold lengths (16,016,071 and 12,720,173 bp), respectively. The pale grey spiral shows the cumulative scaffold count on a log scale with white scale lines showing successive orders of magnitude. The blue and pale-blue area around the outside of the plot shows the distribution of GC, AT and N percentages in the same bins as the inner plot. A summary of complete, fragmented, duplicated and missing BUSCO genes in the metazoa_odb10 set is shown in the top right. An interactive version of this figure is available at https://blobtoolkit.genomehubs.org/view/Candelabrum_cocksii/dataset/GCA_963930725.1/snail.

**Figure 3.**
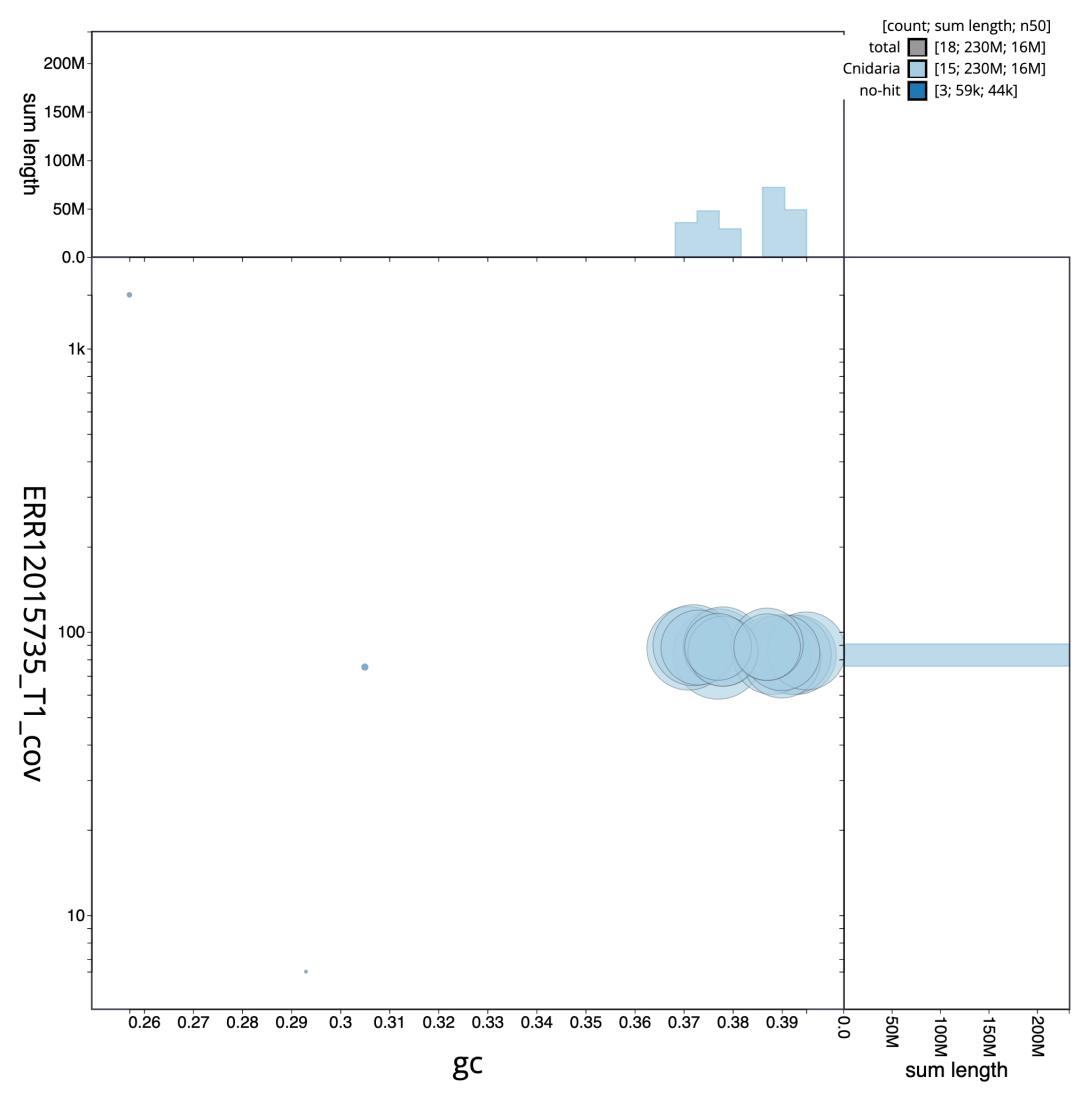
Genome assembly of *Candelabrum cocksii*, jhCanCock6.1: BlobToolKit GC-coverage plot. Sequences are coloured by phylum. Circles are sized in proportion to sequence length. Histograms show the distribution of sequence length sum along each axis. An interactive version of this figure is available at https://blobtoolkit.genomehubs.org/view/Candelabrum_cocksii/dataset/GCA_963930725.1/blob.

**Figure 4.**
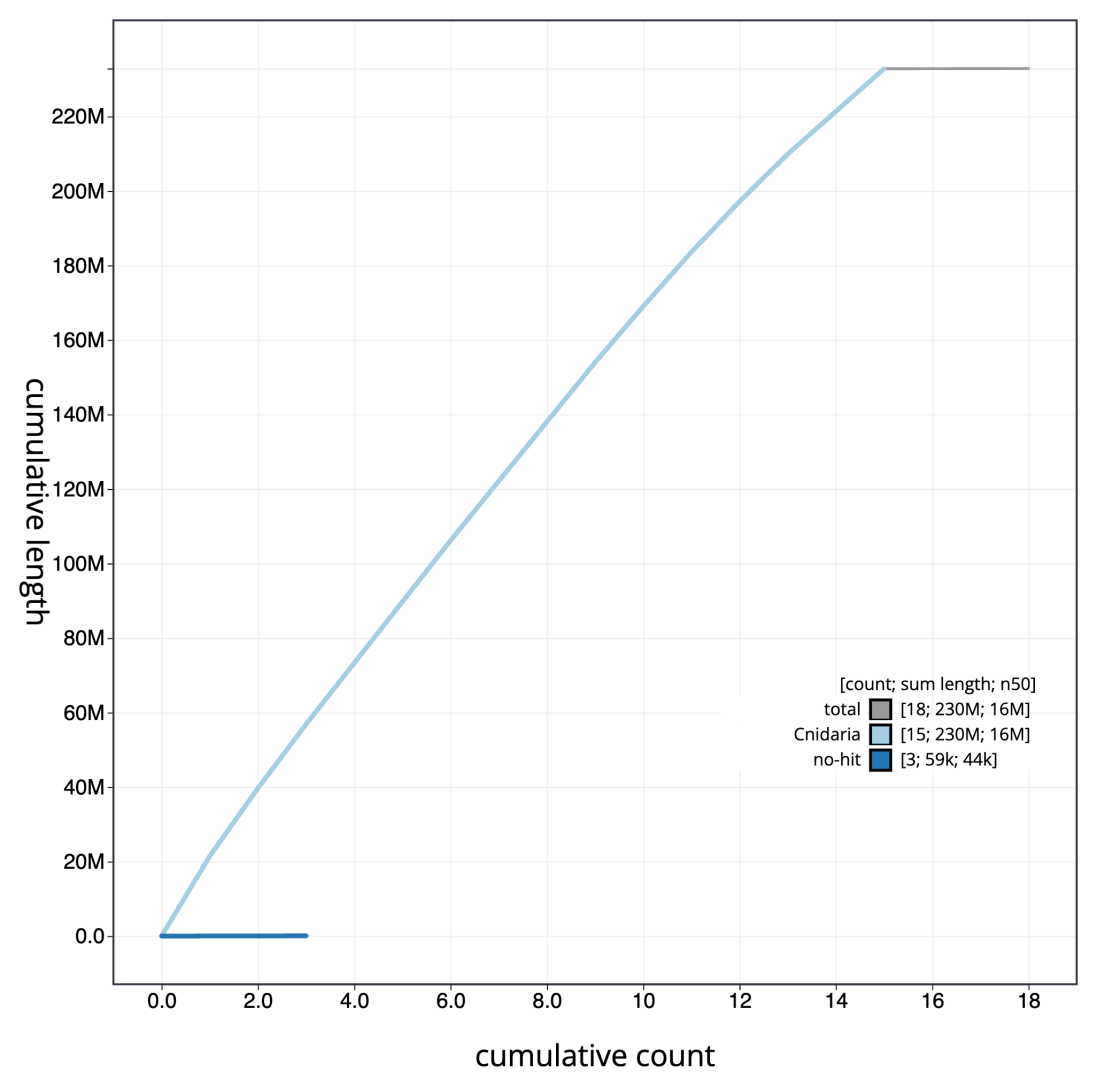
Genome assembly of *Candelabrum cocksii* jhCanCock6.1: BlobToolKit cumulative sequence plot. The grey line shows cumulative length for all sequences. Coloured lines show cumulative lengths of sequences assigned to each phylum using the buscogenes taxrule. An interactive version of this figure is available at https://blobtoolkit.genomehubs.org/view/Candelabrum_cocksii/dataset/GCA_963930725.1/cumulative.

**Figure 5.**
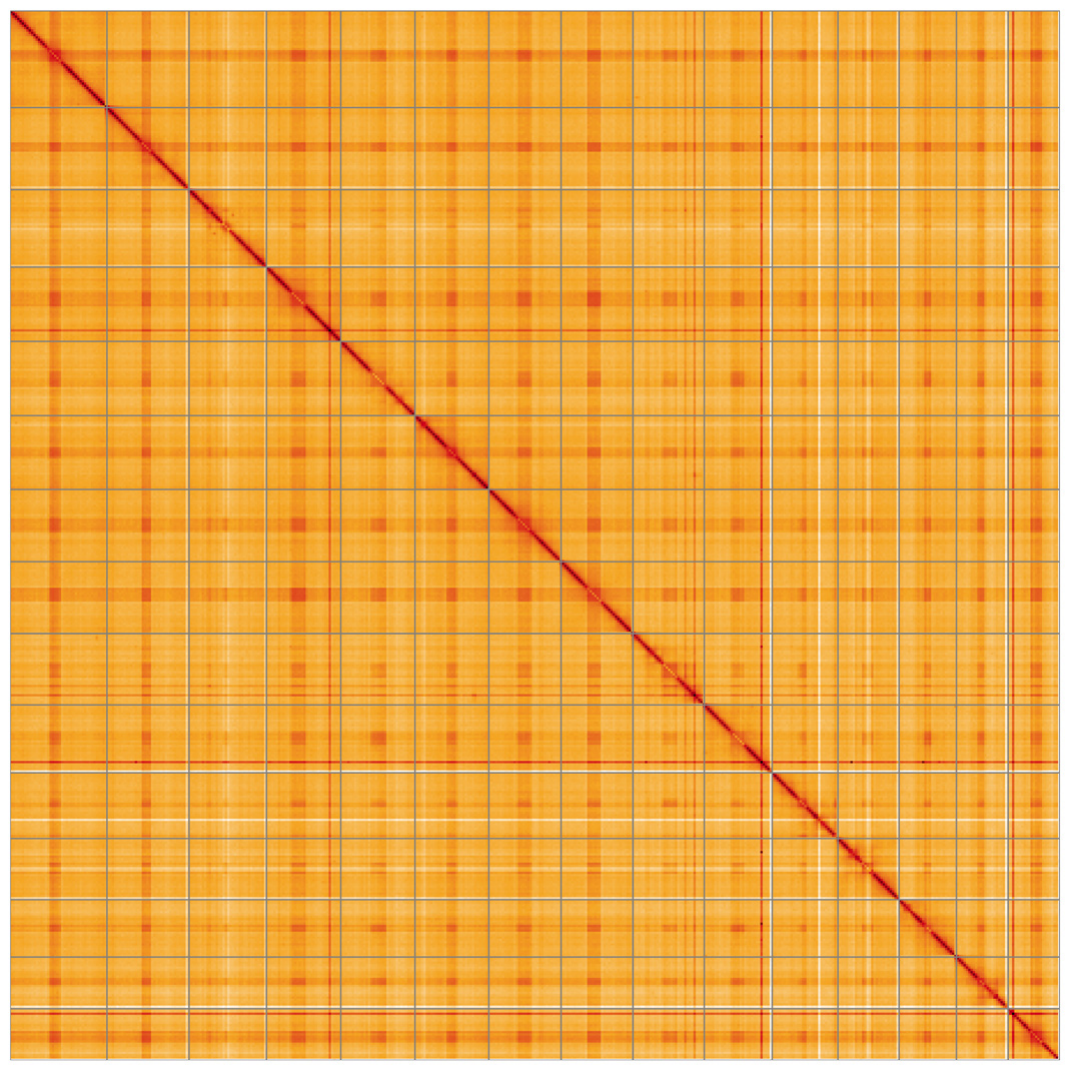
Genome assembly of *Candelabrum cocksii* jhCanCock6.1: Hi-C contact map of the jhCanCock6.1 assembly, visualised using HiGlass. Chromosomes are shown in order of size from left to right and top to bottom. An interactive version of this figure may be viewed at https://genome-note-higlass.tol.sanger.ac.uk/l/?d=NB8bICa7RPaNdK18JGM_2Q.

The final assembly has a total length of 232.9 Mb in 17 sequence scaffolds with a scaffold N50 of 16.0 Mb ( Table 1). The snail plot in Figure 2 provides a summary of the assembly statistics, while the distribution of assembly scaffolds on GC proportion and coverage is shown in Figure 3. The cumulative assembly plot in Figure 4 shows curves for subsets of scaffolds assigned to different phyla. Most (99.97%) of the assembly sequence was assigned to 15 chromosomal-level scaffolds. Chromosome-scale scaffolds confirmed by the Hi-C data are named in order of size ( Figure 5; Table 2). While not fully phased, the assembly deposited is of one haplotype. Contigs corresponding to the second haplotype have also been deposited. The mitochondrial genome was also assembled and can be found as a contig within the multifasta file of the genome submission.

**Table 1.** Genome data for *Candelabrum cocksii*, jhCanCock6.1.

Project accession data		
Assembly identifier	jhCanCock6.1	
Species	*Candelabrum cocksii*	
Specimen	jhCanCock6	
NCBI taxonomy ID	264050	
BioProject	PRJEB65672	
BioSample ID	PacBio long read DNA sequencing: jhCanCock6 whole organism SAMEA13853468 Hi-C scaffolding: jhCanCock1: whole organism SAMEA13853463 RNA sequencing: jhCanCock9 whole organism SAMEA13853471	
Assembly metrics ^*^		*Benchmark*
Consensus quality (QV)	60.5	*≥ 50*
*k*-mer completeness	100.0%	*≥ 95%*
BUSCO ^**^	C:88.1%[S:87.2%,D:0.9%],F:4.7%,M:7.2%,n:954	*C ≥ 95%*
Percentage of assembly mapped to chromosomes	99.97%	*≥ 95%*
Sex chromosomes	None	*localised homologous pairs*
Organelles	Mitochondrial genome: 14.55 kb	*complete single alleles*
Raw data accessions		
PacificBiosciences Sequel IIe	ERR12015735	
Hi-C Illumina	ERR12035249, ERR12035248	
PolyA RNA-Seq Illumina	ERR12035250	
Genome assembly		
Assembly accession	GCA_963930725.1	
*Accession of alternate haplotype*	GCA_963930785.1	
Span (Mb)	232.9	
Number of contigs	135	
Contig N50 length (Mb)	2.8	
Number of scaffolds	17	
Scaffold N50 length (Mb)	16.0	
Longest scaffold (Mb)	21.46	

* Assembly metric benchmarks are adapted from column VGP-2020 of “Table 1: Proposed standards and metrics for defining genome assembly quality” from Rhie *et al.* (2021).

** BUSCO scores based on the metazoa_odb10 BUSCO set using version 5.4.3. C = complete [S = single copy, D = duplicated], F = fragmented, M = missing, n = number of orthologues in comparison. A full set of BUSCO scores is available at https://blobtoolkit.genomehubs.org/view/Candelabrum_cocksii/dataset/GCA_963930725.1/busco.

**Table 2.** Chromosomal pseudomolecules in the genome assembly of *Candelabrum cocksii*, jhCanCock6.

INSDC accession	Chromosome	Length (Mb)	GC%
OZ005664.1	1	21.46	37.5
OZ005665.1	2	18.22	37.0
OZ005666.1	3	17.18	37.0
OZ005667.1	4	16.48	39.5
OZ005668.1	5	16.45	39.0
OZ005669.1	6	16.38	38.0
OZ005670.1	7	16.02	39.0
OZ005671.1	8	15.95	39.0
OZ005672.1	9	15.84	39.5
OZ005673.1	10	15.04	39.0
OZ005674.1	11	14.6	37.5
OZ005675.1	12	13.56	38.5
OZ005676.1	13	12.72	38.0
OZ005677.1	14	11.49	37.5
OZ005678.1	15	11.43	38.5
OZ005679.1	MT	0.01	25.0

The estimated Quality Value (QV) of the final assembly is 60.5 with *k*-mer completeness of 100.0%, and the assembly has a BUSCO v 5.4.3 completeness of 88.1% (single = 87.2%, duplicated = 0.9%), using the metazoa_odb10 reference set ( *n* = 954).

Metadata for specimens, barcode results, spectra estimates, sequencing runs, contaminants and pre-curation assembly statistics are given at https://links.tol.sanger.ac.uk/species/264050.

## Methods

### Sample acquisition and nucleic acid extraction

Specimens of adult *Candelabrum cocksii* were collected from Hannafore Point, Looe, Cornwall, UK (latitude 50.34, longitude –4.45) on 2021-04-26. The specimens were collected and identified by Patrick Adkins and Rob Mrowicki (Marine Biological Association) and preserved by freezing in liquid nitrogen. One individual (specimen ID MBA-210426-001F, ToLID jhCanCock6) was used for PacBio DNA sequencing, another for Hi-C sequencing (specimen ID MBA-210426-001A, ToLID jhCanCock1), and a third for RNA sequencing (specimen ID MBA-210426-001I, ToLID jhCanCock9).

The workflow for high molecular weight (HMW) DNA extraction at the Wellcome Sanger Institute (WSI) Tree of Life Core Laboratory includes a sequence of core procedures: sample preparation; sample homogenisation, DNA extraction, fragmentation, and clean-up. In sample preparation, the jhCanCock6 sample was weighed and dissected on dry ice ( Jay *et al.*, 2023). The tissue was homogenised using a PowerMasher II tissue disruptor ( Denton *et al.*, 2023a).

HMW DNA was extracted using the Manual MagAttract v1 protocol ( Strickland *et al.*, 2023b). DNA was sheared into an average fragment size of 12–20 kb in a Megaruptor 3 system with speed setting 30 ( Todorovic *et al.*, 2023). Sheared DNA was purified by solid-phase reversible immobilisation ( Strickland *et al.*, 2023a): in brief, the method employs a 1.8X ratio of AMPure PB beads to sample to eliminate shorter fragments and concentrate the DNA. The concentration of the sheared and purified DNA was assessed using a Nanodrop spectrophotometer and Qubit Fluorometer and Qubit dsDNA High Sensitivity Assay kit. Fragment size distribution was evaluated by running the sample on the FemtoPulse system.

RNA was extracted from tissue of jhCanCock9 in the Tree of Life Laboratory at the WSI using the RNA Extraction: Automated MagMax™ *mir*Vana protocol ( do Amaral *et al.*, 2023). The RNA concentration was assessed using a Nanodrop spectrophotometer and a Qubit Fluorometer using the Qubit RNA Broad-Range Assay kit. Analysis of the integrity of the RNA was done using the Agilent RNA 6000 Pico Kit and Eukaryotic Total RNA assay.

Protocols developed by the WSI Tree of Life laboratory are publicly available on protocols.io ( Denton *et al.*, 2023b).

### Sequencing

Pacific Biosciences HiFi circular consensus DNA sequencing libraries were constructed according to the manufacturers’ instructions. Poly(A) RNA-Seq libraries were constructed using the NEB Ultra II RNA Library Prep kit. DNA and RNA sequencing was performed by the Scientific Operations core at the WSI on Pacific Biosciences Sequel IIe (HiFi) and Illumina NovaSeq 6000 (RNA-Seq) instruments. Hi-C data were also generated from tissue of jhCanCock1 using the Arima v2 kit. The Hi-C sequencing was performed using paired-end sequencing with a read length of 150 bp on the Illumina NovaSeq 6000 instrument.

### Genome assembly and curation

Assembly was carried out with Hifiasm ( Cheng *et al.*, 2021) and haplotypic duplication was identified and removed with purge_dups ( Guan *et al.*, 2020). The assembly was then scaffolded with Hi-C data ( Rao *et al.*, 2014) using YaHS ( Zhou *et al.*, 2023). The assembly was checked for contamination and corrected using the TreeVal pipeline ( Pointon *et al.*, 2023). Manual curation was performed using JBrowse2 ( Diesh *et al.*, 2023), HiGlass ( Kerpedjiev *et al.*, 2018) and PretextView ( Harry, 2022). The mitochondrial genome was assembled using MitoHiFi ( Uliano-Silva *et al.*, 2023), which runs MitoFinder ( Allio *et al.*, 2020) or MITOS ( Bernt *et al.*, 2013) and uses these annotations to select the final mitochondrial contig and to ensure the general quality of the sequence.

### Evaluation of final assembly

The final assembly was post-processed and evaluated with the three Nextflow ( Di Tommaso *et al.*, 2017) DSL2 pipelines “sanger-tol/readmapping” ( Surana *et al.*, 2023a), “sanger-tol/genomenote” ( Surana *et al.*, 2023b), and “sanger-tol/blobtoolkit” ( Muffato *et al.*, 2024). The pipeline sanger-tol/readmapping aligns the Hi-C reads with bwa-mem2 ( Vasimuddin *et al.*, 2019) and combines the alignment files with SAMtools ( Danecek *et al.*, 2021). The sanger-tol/genomenote pipeline transforms the Hi-C alignments into a contact map with BEDTools ( Quinlan & Hall, 2010) and the Cooler tool suite ( Abdennur & Mirny, 2020), which is then visualised with HiGlass ( Kerpedjiev *et al.*, 2018). It also provides statistics about the assembly with the NCBI datasets ( Sayers *et al.*, 2024) report, computes *k*-mer completeness and QV consensus quality values with FastK and MerquryFK, and a completeness assessment with BUSCO ( Manni *et al.*, 2021).

The sanger-tol/blobtoolkit pipeline is a Nextflow port of the previous Snakemake Blobtoolkit pipeline ( Challis *et al.*, 2020). It aligns the PacBio reads with SAMtools and minimap2 ( Li, 2018) and generates coverage tracks for regions of fixed size. In parallel, it queries the GoaT database ( Challis *et al.*, 2023) to identify all matching BUSCO lineages to run BUSCO ( Manni *et al.*, 2021). For the three domain-level BUSCO lineage, the pipeline aligns the BUSCO genes to the Uniprot Reference Proteomes database ( Bateman *et al.*, 2023) with DIAMOND ( Buchfink *et al.*, 2021) blastp. The genome is also split into chunks according to the density of the BUSCO genes from the closest taxonomically lineage, and each chunk is aligned to the Uniprot Reference Proteomes database with DIAMOND blastx. Genome sequences that have no hit are then chunked with seqtk and aligned to the NT database with blastn ( Altschul *et al.*, 1990). All those outputs are combined with the blobtools suite into a blobdir for visualisation.

All three pipelines were developed using the nf-core tooling ( Ewels *et al.*, 2020), use MultiQC ( Ewels *et al.*, 2016), and make extensive use of the Conda package manager, the Bioconda initiative ( Grüning *et al.*, 2018), the Biocontainers infrastructure ( da Veiga Leprevost *et al.*, 2017), and the Docker ( Merkel, 2014) and Singularity ( Kurtzer *et al.*, 2017) containerisation solutions.

Table 3 contains a list of relevant software tool versions and sources.

**Table 3.** Software tools: versions and sources.

Software tool	Version	Source
BEDTools	2.30.0	https://github.com/arq5x/bedtools2
Blast	2.14.0	ftp://ftp.ncbi.nlm.nih.gov/blast/executables/blast+/
BlobToolKit	4.3.7	https://github.com/blobtoolkit/blobtoolkit
BUSCO	5.4.3 and 5.5.0	https://gitlab.com/ezlab/busco
bwa-mem2	2.2.1	https://github.com/bwa-mem2/bwa-mem2
Cooler	0.8.11	https://github.com/open2c/cooler
DIAMOND	2.1.8	https://github.com/bbuchfink/diamond
fasta_windows	0.2.4	https://github.com/tolkit/fasta_windows
FastK	427104ea91c78c3b8b8b49f1a7d6bbeaa869ba1c	https://github.com/thegenemyers/FASTK
GoaT CLI	0.2.5	https://github.com/genomehubs/goat-cli
Hifiasm	0.16.1-r375	https://github.com/chhylp123/hifiasm
HiGlass	1.11.6	https://github.com/higlass/higlass
HiGlass	44086069ee7d4d3f6f3f0012569789ec138f42b84aa44357826c0b6753eb28de	https://github.com/higlass/higlass
MerquryFK	d00d98157618f4e8d1a9190026b19b471055b22e	https://github.com/thegenemyers/MERQURY.FK
MitoHiFi	2	https://github.com/marcelauliano/MitoHiFi
MultiQC	1.14, 1.17, and 1.18	https://github.com/MultiQC/MultiQC
NCBI Datasets	15.12.0	https://github.com/ncbi/datasets
Nextflow	23.04.0-5857	https://github.com/nextflow-io/nextflow
PretextView	0.2	https://github.com/sanger-tol/PretextView
purge_dups	1.2.3	https://github.com/dfguan/purge_dups
samtools	1.16.1, 1.17, and 1.18	https://github.com/samtools/samtools
sanger-tol/genomenote	1.1.1	https://github.com/sanger-tol/genomenote
sanger-tol/readmapping	1.2.1	https://github.com/sanger-tol/readmapping
Seqtk	1.3	https://github.com/lh3/seqtk
Singularity	3.9.0	https://github.com/sylabs/singularity
TreeVal	1.0.0	https://github.com/sanger-tol/treeval
YaHS	1.1a.2	https://github.com/c-zhou/yahs

### Wellcome Sanger Institute – Legal and Governance

The materials that have contributed to this genome note have been supplied by a Darwin Tree of Life Partner. The submission of materials by a Darwin Tree of Life Partner is subject to the **‘Darwin Tree of Life Project Sampling Code of Practice’**, which can be found in full on the Darwin Tree of Life website here. By agreeing with and signing up to the Sampling Code of Practice, the Darwin Tree of Life Partner agrees they will meet the legal and ethical requirements and standards set out within this document in respect of all samples acquired for, and supplied to, the Darwin Tree of Life Project.

Further, the Wellcome Sanger Institute employs a process whereby due diligence is carried out proportionate to the nature of the materials themselves, and the circumstances under which they have been/are to be collected and provided for use. The purpose of this is to address and mitigate any potential legal and/or ethical implications of receipt and use of the materials as part of the research project, and to ensure that in doing so we align with best practice wherever possible. The overarching areas of consideration are:

●   Ethical review of provenance and sourcing of the material

●   Legality of collection, transfer and use (national and international) 

Each transfer of samples is further undertaken according to a Research Collaboration Agreement or Material Transfer Agreement entered into by the Darwin Tree of Life Partner, Genome Research Limited (operating as the Wellcome Sanger Institute), and in some circumstances other Darwin Tree of Life collaborators.

## Data Availability

European Nucleotide Archive *: Candelabrum cocksii* (a hydroid). Accession number PRJEB65672; https://identifiers.org/ena.embl/PRJEB65672 ( Wellcome Sanger Institute, 2023). The genome sequence is released openly for reuse. The *Candelabrum cocksii* genome sequencing initiative is part of the Darwin Tree of Life (DToL) project. All raw sequence data and the assembly have been deposited in INSDC databases. Raw data and assembly accession identifiers are reported in Table 1.

## Author information

Members of the Marine Biological Association Genome Acquisition Lab are listed here: https://doi.org/10.5281/zenodo.8382513.

Members of the Darwin Tree of Life Barcoding collective are listed here: https://doi.org/10.5281/zenodo.4893703.

Members of the Wellcome Sanger Institute Tree of Life Management, Samples and Laboratory team are listed here: https://doi.org/10.5281/zenodo.10066175.

Members of Wellcome Sanger Institute Scientific Operations: Sequencing Operations are listed here: https://doi.org/10.5281/zenodo.10043364.

Members of the Wellcome Sanger Institute Tree of Life Core Informatics team are listed here: https://doi.org/10.5281/zenodo.10066637.

Members of the Tree of Life Core Informatics collective are listed here: https://doi.org/10.5281/zenodo.5013541.

Members of the Darwin Tree of Life Consortium are listed here: https://doi.org/10.5281/zenodo.4783558.
