# Supplementary material for: The genome sequence of a hydroid, Candelabrum cocksii (Cocks, 1854)
Source: Wellcome Open Res. 2024 Jul 22;9:393. [Version 1] doi: 10.12688/wellcomeopenres.22705.1 (PMC11443189; doi:10.12688/wellcomeopenres.22705.1)
Supplement: Peer review history

# Reviewer report: Reviewer response for version 1

**DOI:** 10.21956/wellcomeopenres.25007.r94931

**Published:** 2024-09-30

**Authors:** Casey Dunn

**Affiliations:** 1 Yale University, New Haven, Connecticut, USA

This is a great contribution to the available cnidarian genomes, and will be very useful to the field. The only comment I have is that the abstract and Hi-C plots indicate 15 chromosomes, but the Genome Sequence report indicates "The final assembly has a total length of 232.9 Mb in 17 sequence scaffolds". Please address this inconsistency explicitly.  Is it in mart due to the mitochondrial genome? Are there scaffolds that do not pass the criteria of chromosomes?

Are sufficient details of methods and materials provided to allow replication by others?

Yes

Is the rationale for creating the dataset(s) clearly described?

Yes

Are the datasets clearly presented in a useable and accessible format?

Yes

Are the protocols appropriate and is the work technically sound?

Yes

Reviewer Expertise:

genomics, cnidarian biology

I confirm that I have read this submission and believe that I have an appropriate level of expertise to confirm that it is of an acceptable scientific standard.

# Reviewer report: Reviewer response for version 1

**DOI:** 10.21956/wellcomeopenres.25007.r95915

**Published:** 2024-09-30

**Authors:** Peter Prentis

**Affiliations:** 1 Queensland University of Technology, Brisbane, Queensland, Australia

The report of The genome sequence of a hydroid, *Candelabrum cocksii *presented in this data note is fine in its current form. The authors have done a good job of presenting the information on the genome sequence and the refinements they used to produce a scaffolded pseudomolecule assembly. The results are logical and easy to understand and the introduction sets out the rationale for the genome sequencing well. I have no further comments.

Are sufficient details of methods and materials provided to allow replication by others?

Yes

Is the rationale for creating the dataset(s) clearly described?

Yes

Are the datasets clearly presented in a useable and accessible format?

Yes

Are the protocols appropriate and is the work technically sound?

Yes

Reviewer Expertise:

Plant and animal genomics.

I confirm that I have read this submission and believe that I have an appropriate level of expertise to confirm that it is of an acceptable scientific standard.
